# The effect of livestock on the physiological condition of roe deer (*Capreolus capreolus*) is modulated by habitat quality

**DOI:** 10.1038/s41598-019-52290-7

**Published:** 2019-11-04

**Authors:** Fernando Horcajada-Sánchez, Gema Escribano-Ávila, Carlos Lara-Romero, Emilio Virgós, Isabel Barja

**Affiliations:** 1 Centro de Investigación, Seguimiento y Evaluación, Parque Nacional de la Sierra de Guadarrama, Ctra. M-604, km 28, 28740 Rascafría, Madrid Spain; 2 Departamento de Ciencias Naturales, Universidad Técnica Particular de Loja, San Cayetano Alto s/n, Marcelino Champagnat, Loja, Ecuador; 3 Área de Biodiversidad y Conservación, Escuela Superior de Ciencias Experimentales y Tecnología, Universidad Rey Juan Carlos, Departamental 1, C/Tulipán s/n, 28933 Móstoles, Madrid Spain; 4 Unidad de Zoología, Departamento de Biología, Facultad de Ciencias, Universidad Autónoma de Madrid. C/Darwin 2. Campus Universitario de Cantoblanco, km 15, 28049 Madrid, Spain; 5 Centro de Investigación en Biodiversidad y Cambio Global (CIBC-UAM), Universidad Autónoma de Madrid, C/Darwin 2, 28049 Madrid, Spain

**Keywords:** Immunological techniques, Ecophysiology

## Abstract

Free-range livestock grazing is a widespread human activity that not only modifies natural vegetation but also leads to interactions with wild ungulates. Most commonly, the interactions between cattle and wild ungulates have been studied with a focus on competition for high-quality forage. However, other mechanisms, such as the risk of parasite infection, might better describe this interaction. We aim to determine whether livestock affect roe deer (*Capreolus capreolus* Linnaeus, 1758) by reducing habitat quality and increasing the probability of infection by shared parasites. We measured noninvasive fecal cortisol metabolites as an indicator of habitat quality as well as the lung nematode larvae burden from the *Dictyocaulus* genus. A higher *Dictyocaulus* larvae load was found in the presence of livestock in pines, and feces collected in winter had a higher parasite load than feces collected in autumn. Additionally, fecal cortisol metabolite levels in the roe deer were affected by the interaction between habitat quality and livestock presence and were higher in the poorest habitat and when living in sympatry with cattle. Our results suggest that physiological stress responses in roe deer were mediated by the habitat type and the presence of competitors. The long-term implications of altered physiological responses such as those demonstrated here should be considered in management strategies for deer.

## Introduction

Habitat loss, fragmentation and degradation are among the main causes of the biodiversity crisis. Because habitat alterations are associated with changes in the spatiotemporal availability of key resources for animals, these changes can produce strong consequences in the life history of animals (e.g., food, cover, refuges and nesting sites)^1^. Free-range livestock grazing, also known as extensive grazing, is one of the most common and widespread human activities throughout the world and greatly modifies the natural vegetation structure and composition. Livestock forage according to their food preferences and plant availability^2^. Generally, they prefer to graze on highly nutritious forbs, generating a high impact in pasture communities, but livestock also consume shrubs, affecting the understory cover^3^.

Even a low level of livestock grazing can contribute to a decrease in food resources for wild ungulates (but could also lead to an increase in or a neutral effect on food resources)^4^. During winter and early spring (*i*.*e*., periods of reduced forage availability) in mountain areas, the effect of livestock grazing can be particularly negative, as this is a period of higher starvation risk for wild ungulates^5^. During these seasons, the diets of livestock and wild ungulates present a strong overlap^4^ (and references therein), leading to food depletion for wild ungulates, which is a form habitat degradation.

Several studies report behavioral variations in wild ungulates, such as the avoidance of areas used by livestock, probably due to competition for food resources, as previously mentioned^6,7^. However, additional mechanisms, such as the reduced risk of parasite infection and transmission, may explain such changes in the behavior of wild ungulates^8^. Parasite transmission rate is a density-dependent process; therefore, for parasites that are shared between wild ungulates and livestock, a larger amount of parasites are expected to be available to infect animals in the presence of livestock. Thus, wild ungulates that feed in areas shared with livestock may face a higher risk of parasitic infection, leading to an increased parasite load^9^. When parasite loads are below a certain threshold and/or animals are in good body condition, increased parasite loads may not affect animals’ capacity of survival or reproduction^10^. However, in other cases, a higher parasite load could dramatically reduce the survival probabilities of the animals^11,12^. Accordingly, to fully understand the relationships of livestock and wild ungulates living in sympatry, not only competition for food resources but also other mechanisms such as parasite infection should be taken into account.

The measurement of stress levels indexed by fecal cortisol metabolites (FCM) has been used in a range of studies to infer environmental factors that may harm fitness, including poor habitat quality or habitat degradation^13^ (for a review). FCM are the result of the activation of the hypothalamic-pituitary-adrenal axis (HPA). When an animal perceives a stressor, its HPA activates, generating a cascading effect from the brain to the adrenal system. This reaction produces an increase in cortisol levels (generally cortisol or corticosterone depending on the species), thereby leading to an increase in glucose availability to help the animals overcome the stressful situation^14–16^. However, prolonged exposure to stressors, such as reduced or poor forage quality, causes increased levels of glucocorticoids in the long term, which leads to ‘chronic stress’^17^. Under these conditions, detrimental effects such as depressed immune responses, reduced reproductive success, suppressed growth, and decreased survival have been observed^15–18^.

According to previous studies, the presence of livestock in the habitat of wild ungulates may lead to increased stress levels as a result of habitat quality reduction that are detectable by means of measuring FCM^19,20^. Additionally, an increased parasite load in wild ungulates could be expected in the presence of livestock owing to higher levels of cross-transmitted parasites. Parasite load itself can be an additional stressor^21^, but increased stress levels may also lead to a poorer performance of the immune function, compromising the capacity of wild ungulates to control parasite infections^22^. Unfortunately, the relationship between stress levels and immune function (that condition parasite load and its consequences on animal health) are far from clear. Detrimental, positive and neutral effects of stress levels on immune function have been found depending on genetic factors, social status and duration of stress^23^ (for a review).

In this study, the aim was to evaluate the effect of livestock on wild ungulate stress levels and parasite load. Among ungulates, the roe deer (*Capreolus capreolus* Linnaeus, 1758) is a typical species in the pine and oak habitats forested in the study area in both the presence and absence of livestock. Thus, we indexed stress levels by measuring FCM and the lung nematode load from the genus *Dictyocaulus*. Cross-transmission between domestic ruminants and roe deer through ingesting larvae of lung nematodes has been reported in previous studies^24^. We performed this study during the forage-limited seasons, and we used two habitats of roe deer that differ in forage quality. Oak forests are a superior habitat compared with pine forests due to their better thermal coverage and more dense and diverse understory (oak forests have a higher abundance of legume and bramble species)^20^. Legumes and brambles are preferred by the roe deer and have been previously related to better body condition^25,26^. In a previous study, several parasite genera were found to be more common in the presence of livestock in pines, which is a poorer habitat than oaks^24^; however, the study did not provide further information on roe deer body condition or physiological status. Here, we first evaluate how the presence of livestock may affect the parasite load of *the Dictyocaulus* genus, many species of which are known to be cross-transmitted between livestock (*Bus taurus*) and roe deer^27,28^. Second, we evaluate how parasite load may affect stress levels of roe deer, in addition to habitat quality and the presence of livestock, in two seasons that vary in their capacity to provide food resources. We hypothesize a higher *Dictyocaulus* larvae load and higher stress levels in the presence of livestock and in the poorer habitat, especially in the pine habitat and during the winter season, which is the most limiting season for the species^20^.

## Results

### Parasite load

**Figure 1:**
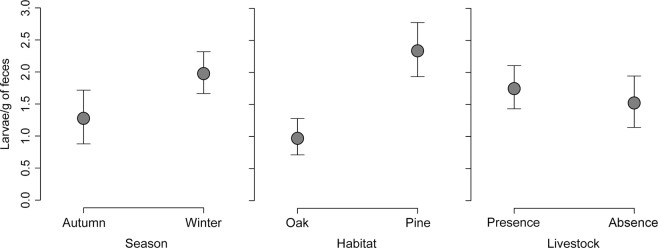
Fecal *Dictyocaulus* larvae concentration in relation to season, habitat type and the presence and absence of livestock.

The overall prevalence of *Dictyocaulus* larvae in the study area was 29%, with a higher prevalence when livestock were present (38 ± 0.07%) than when livestock were absent (20 ± 0.06%). Three models were selected for *Dictyocaulus* larvae load, with Δ AICc < 2 (Table 1, Table S2). The first model included habitat and season, and the second and third models also included livestock and elevation, respectively (Table 1). According to the model estimators, a higher *Dictyocaulus* larvae load was found in the presence of livestock and in pine forest (Table 2a, Table S1, Fig. 1). Feces collected in winter had a higher parasite load than feces collected in autumn (Tables 2a, S1, Fig. 1).

**Table 1:** The highest-ranked linear models using AICc-based model selection for *Dictyocaulus* larvae load. S: season; L: livestock presence/absence; H: Habitat; E: Elevation. Wi: Akaike weight, W+: relative importance of variables. See Table S2 for a complete description of the candidate models considered.

Model	S	L	H	E	adj.R2	df	logLik	AICc	ΔAICc	Wi
6	+		+		0.15	4	−160.069	328.5	0	0.32
8	+	+	+		0.16	5	−159.292	329.1	0.62	0.24
14	+		+	+	0.15	5	−159.808	330.1	1.66	0.14
W+	1	1	0.34	0.2

**Table 2:** Averaged conditional parameters of selected models.

		Estimate	SE
(a)	*Dictyocaulus* larvae abundance		
	intercept	−0.23	0.16
	Habitat-Pine	0.43	0.23
	Season-Winter	0.15	0.18
	Elevation	0.06	0.11
	Livestock-Absent	0.30	0.25
	Livestock-Absent: Habitat-Pine	−0.55	0.36
(b)	FCM concentration		
	intercept	−0.30	0.19
	Habitat-Pine	0.58	0.29
	Livestock-Absent	0.42	0.25

### Physiological stress levels

**Figure 2:**
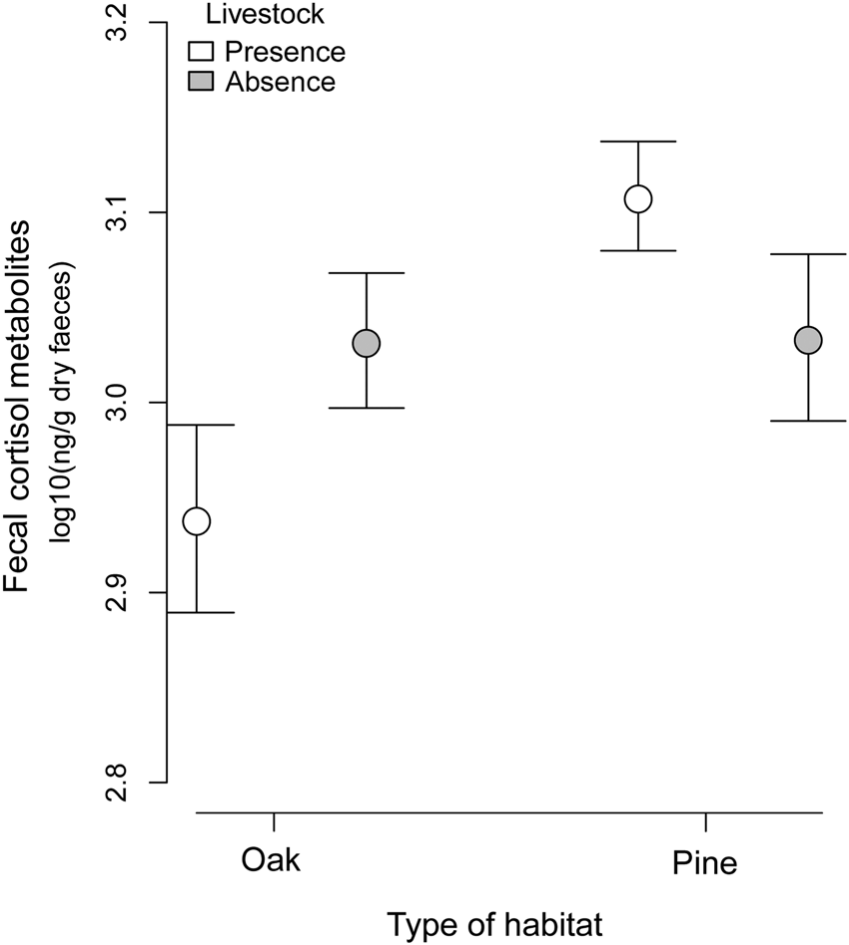
Fecal cortisol metabolite concentrations (ng/g, log-transformed) in relation to habitat type and the presence and absence of livestock.

Stress levels were higher in pine (1331.5 ± 76.6 ng/g dry feces, n = 60) than in oak forests (1098.1 ± 64.2 ng/g dry feces, n = 60), with a significant mean difference (233.3 ± 99.9 *p* < 0.05). According to the multimodel inference results, two models were equally valid (Table 3, Table S3). One model included the variable of habitat, and the other included habitat, livestock and their interaction. Thus, the most relevant variable explaining FCM was habitat, according to the multimodel inference (maximum value of relative importance, W_+_ = 1), followed by livestock and their interaction. Models with elevation, season or parasite load performed poorly (Δ AICc > 2) and were not included in the confidence set of models (Table S3). Overall, the results showed that the highest stress levels were shown in the poorer habitat (pine) and in the presence of livestock (Table 2b and Table S4, Fig. 2), given a body of evidence to verify our hypothesis. Nevertheless, this pattern was not always consistent, since the interaction habitat x livestock was selected. This was because the presence of livestock had opposite effects in oak and pine habitats (Table 2b and Table S4, Fig. 2).

**Table 3:** Highest ranked linear models using AICc-based model selection for FCM concentration. L: livestock presence/absence; H: Habitat. Wi: Akaike weight, W+: relative importance of variables. See Table S3 for information on all the candidate models considered.

Model	G	H	G:H	adj.R2	df	logLik	AICc	ΔAICc	Wi
39	+	+	+	0.10	5	−165.259	341	0	0.147
5		+		0.04	3	−167.514	341.2	0.2	0.133
W+	0.52	1	0.52

## Discussion

We were able to confirm our hypothesis. Our results reveal that the overall prevalence of *Dictyocaulus* parasites was associated with the poorer habitat and the more limiting season, as we predicted. As previously shown by other studies, parasite load was positively related to the presence of livestock, but stress levels were differently affected by the presence of livestock depending on the quality of habitat. In oaks, the superior habitat, roe deer showed lower stress levels in the presence than in the absence of livestock. This is an unexpected result that can be explained by the modifications that the livestock perform that lead to more open habitats that are richer in understory shrubs that are related to roe deer body conditions. Our results suggest that oak habitats are able to sustain roe deer and livestock in sympatry without negative costs in terms of stress levels for the wild species. This pattern could be explained by different processes, but because pine forests have low-quality food resources, individuals can be prone to suffering stress conditions under interspecific competition with cattle.

Regarding the role of habitat, forage quality and livestock in roe deer stress levels, our results add to the available literature reporting differences in fecal cortisol levels for different species and populations due to changes in habitat quality^20,29,30^. In this study, higher cortisol levels in feces were found in pine than in oak forests. This is in line with other findings stating that roe deer preferred habitat types are those mainly composed of open deciduous forests with an abundant and diverse understory (e.g.^25^). Previous literature shows how certain understory plant species such as oak, brambles, hawthorns and *Rosa* spp. are relevant to determining habitat quality for roe deer in terms of deer abundance but also in terms of body condition^26,31,32^. This was also the case in our study, where roe deer inhabiting oak habitats presented lower stress levels than deer in pine habitats, in which the understory was mostly depleted. Pine habitats are located at a higher altitude in the study area (1325.4 ± 128.9 m.a.s.l), with lower minimum temperatures and more freezing periods^20^. These environmental conditions together with a denser canopy that reduces light availability could be enough to explain the differences in understory cover and diversity between the two habitats. In addition, removal of shrub vegetation is performed in this habitat as a forestry management technique to combat fires^33,34^. This has worsened the natural condition of the understory in pine forests. Thus, a combination of environmental conditions and active management to combat fires (pruning, clearing, creating clear firebreaks) that reduces the overall understory may explain the low capacity of pine habitats to provide quality forage to roe deer, leading to increased stress levels, especially in the presence of livestock.

The presence of livestock interacted with habitat quality, leading to a negative impact on the physiological condition of roe deer in the poorest habitats, as we hypothesized. However, the presence of livestock in oak habitats led to lower stress levels, which is hard to explain, considering the expected competition for food resources between roe deer and livestock^35^.

The observed results regarding season further support the important role of habitat quality in determining stress levels in roe deer^20^. Our study was conducted from mid-fall to winter, a period in which food resources for both species are likely to be reduced^4^, especially in pine habitats^20^. These results match previous findings stating that during periods of low food resource availability, dietary overlap between livestock and wild ungulates is more intense and thus leads to higher competition^36,37^.

Livestock presence is positively related to the *Dictyocaulus* larvae burden in roe deer. *Dictyocaulus* larvae in this study were collected from animal feces; therefore, we could not identify the particular parasite species affecting the deer (for such identification, lung tissue with adult parasites and/or molecular analyses are needed). However, cross-transmission of *Dictyocaulus* lung worms in cattle and several cervid species, including roe deer, have been reported experimentally. Additionally, it has been suggested that cervids are an important reservoir for *D*. *viviparus*, the parasite that causes bovine parasitic bronchitis in livestock^27^. It is known that 40% of nematode parasites of roe deer are shared with domestic ungulates, and cattle share over 50% of parasites with wild ungulates, suggesting quite a generalist (*i*.*e*., non-species-specific) relationship between nematode parasites and wild and domestic ungulates^38^.

Common prevalence values of *Dictyocaulus* spp. in roe deer range between 2 and 24%, with typical values of approximately 15%, matching our results for prevalence in the absence of livestock^28,39–42^. However, the prevalence of *Dictyocaulus* in the area with livestock (1.3 cattle/10 ha) was 38%, which is notably higher than the highest prevalence formerly reported. A previous attempt to relate the presence of domestic ungulates to increased *Dictyocaulus* larvae load in shot roe deer failed to find such a relationship. However, the authors of this work acknowledge that this prevalence could be biased, as deer in poor condition were selectively shot, and this could have biased the results^39^. Alternatively, Vazquez *et al*.^40^ detected an increase of 15% in the prevalence of roe deer infected with *Dictyocaulus* larvae related to an increase in host density. Overall, more empirical information is needed to clarify the relationship between livestock, roe deer and *Dictyocaulus*. If we are to understand such a relationship, *Dictyocaulus* geographic distribution, host species and cross-transmission rates at the intra- and interspecific levels should be better known^43^. Interestingly, although the *Dictyocaulus* prevalence detected in our study was higher than that in other studies, we did not find a positive relationship between *Dictyocaulus* load and stress levels. Based on this result, it seems that parasite load, despite being high comparable to that other areas, has not crossed a threshold that would the physiological system of roe deer. Parasites regulate populations in some cases and decimate species in other cases. However, under many environmental conditions, these pathogens simply exist with neither positive nor highly detrimental effects to the host animal^11^. In other studies, corticosterone-parasite interactions are often bidirectional: increased glucocorticoid levels may suppress the immune system, but parasitic infection may increase corticosterone levels either through infection-induced stress or through stress-induced immunosuppression^21^. In our study, animals with higher lung nematode loads had levels of cortisol metabolites similar to those in less-parasitized animals, suggesting that parasites are not imposing a cost on the general body condition of roe deer. A previous study performed by our research team reports the good capacity of roe deer to successfully reproduce in an array of habitat types in the central Iberian Peninsula^20^, which, together with the findings shown here, assist in providing mechanistic evidence for the recent expansion of the roe deer population from the northern to the more southern and xeric areas near the edge of the Iberian Peninsula. Therefore, our results contribute to a large body of already-available evidence showing that roe deer populations in the central Iberian Peninsula are in good physical condition.

In conclusion, the consideration of habitat quality is pivotal to understanding the extent to which human activities may be compatible with the conservation and well-being of wild animal populations, something of particular concern in protected areas. According to our results, oak habitats provide good forage quality for both livestock and deer. In the case of pine forests, the sparse understory (due to natural environmental conditions and additional management to prevent fire) leads to an impoverished habitat that is not able to sustain livestock and roe deer in sympatry without imposing a cost on the physiological condition of wild ungulates. Although the presence of livestock was related to a greater *Dictyocaulus* larvae burden, this did not seem to compromise the physiological condition of roe deer. The presence of livestock may further compromise the fitness of animals depending on individual animal and environmental variables. Overall, the study showed that by studying stress levels together with habitat quality and parasite loads, it is possible to detect incipient human disturbances to wild species. This information is valuable and may help in the adoption of management solutions in natural environments, thereby reducing negative interactions among humans, livestock production, and wild animal populations^44^.

## Methods

### Study area and species studied

**Figure 3:**
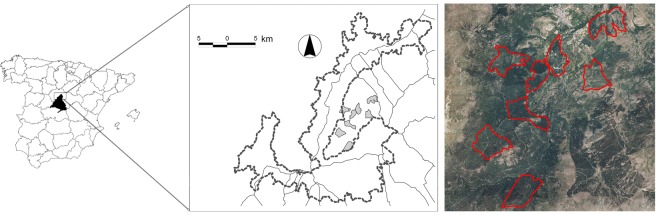
Location of the study area and Sierra de Guadarrama National Park in the central Iberian Peninsula. The locations of the eight plots sampled are shown in gray on the map and red on the orthoimage view. The layout was created with QGis 3.6, https://www.qgis.org/es/site/ and the orthoimage view was derived from PNOA 2010–2013 CC-BY scne.es.

The study was conducted during the nonbreeding period, from October 2009 to March 2010, on the borders of Sierra de Guadarrama National Park, in the central Iberian Peninsula (Fig. 3). The sampled plots were in public forests of wild pine (*Pinus sylvestris* L.) and oak (*Quercus pyrenaica* Willd.). The pine forests occur between 1,200 and 1,900 m a.s.l. and generally have low shrub diversity attributed mainly to forest management for wildfire suppression^33^. The oak forests are predominantly above 1,100 m a.s.l. with an understory of broom (*Cytisus scoparius* L.), willows (*Salix atrocinerea* Brot.), hawthorn (*Crataegus monogyna* Jacq), blackthorn (*Prunus spinosa* L.) and *Rosa* spp.

Previous studies established that the mean roe deer density (individuals/100 ha) in the absence of livestock was 5.9 and 5.4 in pines and oaks, respectively, while in the presence of livestock, it was 4.8 and 4.5 for pine and oak habitats^45,46^. The mean density (roe deer/100 ha) obtained with the linear transects was 5.2 ± 0.96 (CV % = 18.22, 95% CI = 3.67 to 7.87); transect data were analyzed using Distance 6.0^47^.

The free-ranging livestock regime included only cows (13 individuals/100 ha, National Office of Statistic 2009), which were equally abundant in both habitats. No farms with permanent grazing infrastructure were present. The only wild ungulate present, in addition to the roe deer, was the wild boar (*Sus scrofa* Linnaeus, 1758), at low densities (<3 individuals/100 ha in pine and oak forests) throughout the study area.

### Collection of fecal samples

A total of 120 fresh fecal samples from roe deer were collected from a total of eight plots with an area ranging from 100 to 168 ha (139 ± 8.4 ha), 30 in pine with livestock, 30 in pine without livestock, and the same in the oak forest, from October 2009 to March 2010 (Table 4). Fresh feces were characterized by a moist layer of bright green mucus, were soft to the touch and showed no signs of dehydration^20^. Four of the plots were established in oak and four in pine forests. Two plots in oak and two in pine had the presence of cattle. The area for the study plots was chosen according to the roe deer home range and their behavior in order to maximize the number of animals sampled and, in this way, to minimize the effects of anonymous sampling (over- or underrepresentation of the same individuals to the detriment of others^48^). Thus, the sampled plots (oak, 134 ± 9.8 ha; pine, 143 ± 16.1 ha) were below the average size of the roe deer home range for both males and females, especially in forested areas with low densities, such as those in the study area^49,50^. Females overlap their home ranges; therefore, several females and their offspring can share the same territory^51^. The groups with the highest number of roe deer, including the males, were observed during autumn and winter^52^. Therefore, based on this evidence and the species densities in the habitats of the study area^46^, we estimated that the plots would have maintained a minimum of 10 individuals each.

**Table 4:** Collection of roe deer fecal samples by season and habitat.

		Winter	Autumn	Total
Habitat	Oak	33 (27.5%)	27 (22,5%)	60 (50%)
	Pine	33 (27.5%)	27 (22,5%)	60 (50%)
Total		66 (55%)	54 (45%)	120 (100%)

Huber *et al*.^48^ found no significant differences in FCM levels between known and anonymous samples from a population. Although these authors indicate that the technique is reliable, they recommended the use of linear transects to maximize the sampling area and the number of animals sampled. We followed this methodology in each of the four plots sampled in each habitat type. In each plot, 15 transects were established following pathways typically used by the species, and one sample was collected for each transect. Roe deer activity increases around sunrise^53,54^; hence, all transects were surveyed at 8 a.m. to increase the probability of detecting fresh feces. Of 120 fecal samples collected, 30 were collected in pine with livestock and 30 in pine without livestock, and the same collection effort was made in oak forest. From each fresh scat, 12 pellets were collected by means of a gloved hand and considered a sample. The samples were divided into subsamples of six pellets for analysis of cortisol metabolites and the other six for coprological analysis of lung nematodes. Samples in the field were stored in a portable cooler at 4 °C until transfer to the lab. We homogenized the entire fecal mass in the samples collected to analyze cortisol metabolites following Barja *et al*.^55^.

### Extraction and enzyme immunoassay

The samples for hormonal analysis were maintained at −20 °C until being assayed. For the measurement of stress levels indexed by FCM, cortisol metabolites were extracted from fecal samples following the procedure described by Escribano *et al*.^20^. In brief, fecal samples were dried at 39 °C until stable weight was obtained. Subsamples (0.7 g) were placed in assay tubes with 2.5 ml of phosphate buffer and 2.5 ml of methanol and shaken for 16 h. The supernatant was centrifuged at 4000 g for 30 min, and then the pellet was discarded and the fecal extracts were stored at −20 °C until being assayed. A cortisol commercial kit (EIA; DRG Instruments GMBH 211, Marbug, Germany) was used to perform the enzyme immunoassays. This methodology has been previously used and validated (ACTH challenge, parallelism test, intra- and interassay coefficients of variation) to monitor the stress levels in roe deer (see^20^). FCM levels were expressed in ng/g dry feces.

### Coprological analysis

The prevalence of lung nematodes such as *Dictyocaulus* depends upon weather conditions and is directly related to the grazing season^56^. The samples used for parasitological analysis were stored at 4 °C until processing. The detection of lung nematode larvae of *Dictyocaulus* spp. in the fecal samples was performed by means of the Baermann method to assess the number of first-stage larvae (L-1) per gram (lpg)^57^ using 0.92 ± 0.4 g of fecal matter. Microscopic counts were carried out in McMaster chambers following the MAFF^58^ protocol. The results are expressed in larvae/g of feces.

### Statistical analyses

To minimize observer bias, we used blind methods when all fecal samples were collected and physiological levels were analyzed. Thirty-three samples of the 120 were in poor condition for parasite analysis, as they were already frozen when found in the field (owing to the low temperatures in the early morning in winter). To address missing data, we implemented the nonparametric *missForest* imputation method, which imputes missing values using random forest predictions^59^. We used the box-plot method to detect outliers in the original database. Namely, an observation was considered an outlier if it fell outside the 1.5*IQR limits, where IQR, the ‘interquartile range’ is the difference between the 75th and 25th quartiles. We subsequently imputed outliers following the same methodology as for the imputation of missing values.

We used linear models (LMs) to determine whether *Dictyocaulus* larvae load and FCM levels (dependent variables) varied in relation to habitat type (pine forest/oak forest), livestock (presence/absence) and the interaction between these factors. We also included the elevation (m.a.s.l) and the season in which the fecal samples were collected as predictors in the model to control for temporal and spatial variation. In models fitted for FCM levels, we also included *Dictyocaulus* larvae load as a predictor variable for evaluating whether the parasite load itself represents an additional stressor for the studied roe deer populations. The response variables were transformed to meet the assumptions of normality and homoscedasticity: FCM was log10-transformed and *Dictyocaulus* larvae load was power-transformed using the Yeo–Johnson transformation^60^. The Yeo-Johnson transformation has properties similar to those of the Box–Cox transformation but allows for zero and negative values. Model residuals were checked graphically for normality and homogeneity of variances using diagnostic plots^61^. All continuous variables were centered and scaled to a mean of zero ± 1 standard deviation to allow the direct comparisons of the regression coefficients^62^. We performed a model selection procedure with all possible combinations of explanatory variables and evaluated the best model by means of AICc with a threshold of ΔAICc > 2^63^. The candidate model set contained 20 and 40 models for *Dictyocaulus* larvae load and FCM levels, respectively (Tables S2, S3). If more than one single model was selected, we evaluated the set of confidence models (those within ΔAICc > 2) and performed a multimodel inference approach based on model averaging. Statistical analyses were conducted in R^64^. The missForest approach was implemented in the ‘missForest’ package in R^59^, while variable transformations and standardization were implemented using the R package ‘bestNormalize’^65^.

## Supplementary information

Supplementary tables

Dataset 1.

## Data Availability

The datasets generated during and/or analyzed during the current study are available in the Supplementary Information File.
