# Supplementary material for: The effect of livestock on the physiological condition of roe deer (Capreolus capreolus) is modulated by habitat quality
Source: Sci Rep. 2019 Nov 4;9:15953. doi: 10.1038/s41598-019-52290-7 (PMC6828671; doi:10.1038/s41598-019-52290-7)
Supplement: Supplementary file 1 — Supplementary tables [file 41598_2019_52290_MOESM1_ESM.pdf]

The effect of livestock on the physiological condition of roe deer (*Capreolus capreolus*) is modulated by habitat quality

Fernando Horcajada-Sánchez [1*], Gema Escribano-Ávila [2], Carlos Lara-Romero [3], Emilio Virgós [3] and Isabel Barja [4, 5]

[1]Centro de Investigación, Seguimiento y Evaluación, Parque Nacional de la Sierra de Guadarrama, Ctra. M-604, km 28, 28740 Rascafría, Madrid, Spain. Email (FHS): fernando.horcajada@pnsg.es

[2]Departamento de Ciencias Naturales. Universidad Técnica Particular de Loja, San Cayetano Alto s/n, Marcelino Champagnat, Loja, Ecuador. Email (GEA): gema.escribano.avila@gmail.com

[3]Área de Biodiversidad y Conservación, Escuela Superior de Ciencias Experimentales y Tecnología, Universidad Rey Juan Carlos, Departamental 1, C/ Tulipán s/n, E-28933 Móstoles, Madrid, Spain. Email (CLR): carlos.lara.romero@gmail.com; Email (EV): emilio.virgos@urjc.es

[4]Unidad de Zoología, Departamento de Biología, Facultad de Ciencias, Universidad Autónoma de Madrid. C/Darwin 2. Campus Universitario de Cantoblanco, km 15, 28049 Madrid, Spain. Email (IB): isabel.barja@uam.es

[5]Centro de Investigación en Biodiversidad y Cambio Global (CIBC-UAM), Universidad Autónoma de Madrid, C/ Darwin 2, 28049 Madrid, Spain: isabel.barja@uam.es

[*]corresponding author (FHS): fernando.horcajada@pnsg.es

Supplementary tables

Table S1. Mean *Dictyiocaulus* larvae load (Larvae/g of feces) in the two studied habitat and seasons and in the presence and absence of livestock.

Season	Habitat	Livestock	Mean (Larvae/g)	SE
Autumn	Oak	Present	0.19	0.09
		Absent	0.01	0.01
	Pine	Present	1.82	0.62
		Absent	3.05	1.31
Winter	Oak	Present	2.04	0.69
		Absent	1.39	0.66
	Pine	Present	2.52	0.72
		Absent	1.89	0.46

Table S2. Multi model inference results for *Dictyocaulus* larvae load. The table also shows model number (Ni), maximised log-likelihood function (log(L)), number of estimated parameters (*df*); AICc differences (ΔAICc) and Akaike weights (wi). S: season; L: livestock presence/absence; H: Habitat; E: Elevation; Wi: Akaike weight.

Ni	Int	S	L	H	E	L:H	df	logLik	AICc	ΔAICc	Wi
6	+	+		+			4	-160.069	328.50	0	0.32
8	+	+	+	+			5	-159.292	329.10	0.62	0.24
14	+	+		+	+		5	-159.808	330.10	1.66	0.14
16	+	+	+	+	+		6	-159.039	330.80	2.33	0.10
18	+	+	+	+		+	6	-159.212	331.20	2.68	0.09
20	+	+	+	+	+	+	7	-158.994	333.00	4.5	0.03
2	+	+					3	-163.606	333.40	4.93	0.03
4	+	+	+				4	-162.873	334.10	5.61	0.02
10	+	+			+		4	-163.245	334.80	6.35	0.01
12	+	+	+		+		5	-162.497	335.50	7.03	0.01
7	+		+	+			4	-165.416	339.20	10.69	0.00
5	+			+			3	-166.589	339.40	10.9	0.00
15	+		+	+	+		5	-164.893	340.30	11.83	0.00
13	+			+	+		4	-166.041	340.40	11.94	0.00
17	+		+	+		+	5	-165.343	341.20	12.73	0.00
19	+		+	+	+	+	6	-164.865	342.50	13.99	-
3	+		+				3	-168.659	343.50	15.04	-
1	+						2	-169.771	343.60	15.16	-
11	+		+		+		4	-168.514	345.40	16.89	-
9	+				+		3	-169.643	345.50	17.01	-

Table S3. Multi model inference results for FCM levels. The table also shows model number (Ni), maximised log-likelihood function (log(L)), number of estimated parameters (*df*); AICc differences (ΔAICc) and Akaike weights (wi). S: season; L: livestock presence/absence; H: Habitat; E: Elevation, D: *Dictyocaulus* larvae load; Wi: Akaike weight.

Ni	Int	S	L	H	D	E	L:H	df	logLik	AICc	ΔAICc	Wi
39	+		+	+			+	5	- 165.26	341.00	-	0.15
5	+			+				3	- 167.51	341.20	0.19	0.13
40	+	+	+	+			+	6	- 165.19	343.10	2.08	0.05
21	+			+		+		4	- 167.45	343.30	2.21	0.05
55	+		+	+		+	+	6	- 165.26	343.30	2.22	0.05
47	+		+	+	+		+	6	- 165.26	343.30	2.22	0.05

6	+	+		+				4	-	167.46	343.30	2.22	0.05
7	+		+	+				4	-	167.49	343.30	2.27	0.05
13	+			+	+			4	-	167.50	343.40	2.31	0.05
1	+							2	-	169.77	343.60	2.60	0.04
17	+					+		3	-	168.73	343.70	2.63	0.04
22	+	+		+		+		5	-	167.38	345.30	4.25	0.02
8	+	+	+	+				5	-	167.42	345.40	4.32	0.02
56	+	+	+	+		+	+	7	-	165.19	345.40	4.33	0.02
23	+		+	+		+		5	-	167.43	345.40	4.33	0.02
48	+	+	+	+	+		+	7	-	165.19	345.40	4.34	0.02
14	+	+		+	+			5	-	167.44	345.40	4.36	0.02
29	+			+	+	+		5	-	167.44	345.40	4.37	0.02
15	+		+	+	+			5	-	167.48	345.50	4.43	0.02
63	+		+	+	+	+	+	7	-	165.26	345.50	4.47	0.02
18	+	+				+		4	-	168.63	345.60	4.56	0.02
9	+				+			3	-	169.71	345.60	4.58	0.02
2	+	+						3	-	169.72	345.60	4.60	0.02
3	+		+					3	-	169.74	345.70	4.65	0.01
19	+		+			+		4	-	168.71	345.80	4.72	0.01
25	+				+	+		4	-	168.71	345.80	4.73	0.01
24	+	+	+	+		+		6	-	167.35	347.40	6.39	0.01
30	+	+		+	+	+		6	-	167.36	347.50	6.43	0.01
16	+	+	+	+	+			6	-	167.40	347.50	6.50	0.01
31	+		+	+	+	+		6	-	167.42	347.60	6.53	0.01
64	+	+	+	+	+	+	+	8	-	165.19	347.70	6.63	0.01
10	+	+			+			4	-	169.66	347.70	6.63	0.01
11	+		+		+			4	-	169.68	347.70	6.65	0.01
4	+	+	+					4	-	169.68	347.70	6.66	0.01
20	+	+	+			+		5	-	168.59	347.70	6.67	0.01
26	+	+			+	+		5	-	168.62	347.80	6.72	0.01
27	+		+		+	+		5	-	168.69	347.90	6.86	0.01
32	+	+	+	+	+	+		7	-	167.33	349.70	8.61	0.00
12	+	+	+		+			5	-	169.63	349.80	8.73	0.00
28	+	+	+		+	+		6	-	168.58	349.90	8.87	0.00

Table S4. Mean fecal cortisol concentration (ng/g) metabolites in the two studied habitats in the presence and absence of livestock.

Habitat	Livestock	Mean (ng/g)	SE
Oak	Present	1020.7	96.85
	Absent	1175.5	83.68
Pine	Present	1364.01	81.99
	Absent	1298.92	130.59
